# Nicotine Has a Therapeutic Window of Effectiveness in a *Drosophila melanogaster* Model of Parkinson's Disease

**DOI:** 10.1155/2022/9291077

**Published:** 2022-07-08

**Authors:** Brady T. Mannett, Braden C. Capt, Krista Pearman, Lori M. Buhlman, John M. VandenBrooks, Gerald B. Call

**Affiliations:** ^1^Arizona College of Osteopathic Medicine, Midwestern University, Glendale, AZ 85308, USA; ^2^OSF Neurology at University of Illinois, Peoria, IL 61637, USA; ^3^Department of Pharmacology, College of Graduate Studies, Midwestern University, Glendale, AZ 85308, USA; ^4^Biomedical Science Program, College of Graduate Studies, Midwestern University, Glendale, AZ 85308, USA; ^5^Department of Physiology, College of Graduate Studies, Midwestern University, Glendale, AZ 85308, USA

## Abstract

Strong epidemiological evidence and studies in models of Parkinson's disease (PD) suggest that nicotine may be therapeutically beneficial in PD patients. However, a number of clinical trials utilizing nicotine in PD patients have had mixed results, indicating that either nicotine is not beneficial in PD patients, or an important aspect of nicotine therapy was absent. We hypothesized that nicotine must be administered early in the adult fly life in order to have beneficial effects. We show that continuous early nicotine administration improves both climbing and flight deficiencies present in homozygous *park*^*25*^ mutant PD model *Drosophila melanogaster*. Using a new climbing assay, we identify several climbing deficiencies in this PD model that are improved or rescued by continuous nicotine treatment. Amongst these benefits, it appears that nicotine improves the ability of the *park*^*25*^ flies to descend the climbing vial by being able to climb down more. In support of our hypothesis, we show that in order for nicotine benefits on climbing and flight to happen, nicotine administration must occur in a discrete time frame following adult fly eclosure: within one day for climbing or five days for flight. This therapeutic window of nicotine administration in this PD model fly may help to explain the lack of efficacy of nicotine in human clinical trials.

## 1. Introduction

Parkinson's disease (PD) is the second most common neurodegenerative disease, after Alzheimer's disease, with clinical symptoms including resting hand tremor, an impaired sense of balance, dyskinesia, and an unsteady or impaired gait [1, 2]. PD occurs in both a familial or genetic form such as Autosomal Recessive Juvenile Parkinsonism (AR-JP), and the more common idiopathic or sporadic, adult onset form [1]. Within the last 20 years, the Parkin protein, the *PRKN* gene product, in humans and other model organisms has been studied extensively. Parkin is an E3 ubiquitin ligase and is functionally linked to Phosphotensin-induced kinase 1 (PINK1) [3, 4]. These proteins are involved in mitophagy, a process that healthy mitochondria undergo under normal conditions [5–7]. Therefore, the downstream effects of a mutated *PRKN* gene lead to a defect in the function or morphology of the mitochondria and this contributes ultimately to the development of AR-JP [8].

Many genetic homologs exist between *Drosophila melanogaster*, the fruit fly, and vertebrates, making it a valuable tool for the study of human diseases [9–11]. Indeed, *Drosophila* has been extremely valuable for understanding the function of PD-causing genes [12]. Mutation of the *Drosophila* ortholog of the *PRKN* gene, *parkin* (*park*), leads to a tenable model of PD that has many similarities to PD patients: selective loss of dopaminergic neurons, decreased motor function, loss of olfaction, reduced lifespan, mitochondrial dysfunction, and others [13–17]. Another use of *Drosophila* PD models has been to identify potential therapeutics for PD, including, but not limited to, nicotine, acteoside, gastrodin, and other nutraceuticals [13, 18–21].

There is currently no cure for PD, but rather only management of disease symptoms. Dopamine replacement therapy, through the use of levodopa or carbidopa, and deep brain stimulation, are currently the standard of care for PD patients. However, interesting epidemiological evidence suggest that tobacco use coincides with lower incidences of PD [22, 23]. The paramount therapeutic agent in tobacco products is considered to be nicotine. Nicotine prevents significant loss of striatal dopamine, and nicotine exposure has a protective role against neuronal insults in experimental models [24, 25]. Indeed, previous research in our laboratory showed that when heterozygous *park*^*25*^ flies were treated with nicotine, they demonstrated improvements in climbing, flight, lifespan, and olfaction [13]. More recently, nicotine treatment in another *Drosophila* PD model also suppressed PD-like phenotypes [20].

Unfortunately, nicotine therapy of PD patients in a small number of clinical trials has yielded mixed results. In one study, PD patients treated with nicotine scored lower in motor and cognitive tasks than controls [26]. Another study found that when transdermal nicotine patches, in addition to the conventional drugs, were administered for three weeks to sporadic PD patients, no affective or motor symptoms were alleviated [27]. Yet another study found that the use of these same nicotine patches worsened the patient's symptoms (e.g., patients showed poorer performance in various motor tasks and cognition performance) [28]. These results, especially with regard to the epidemiological studies, hinted that there may be an issue with previous human PD nicotine clinical trials. One possible factor that has been postulated is the timing of the nicotine therapy [29]. Unlike long-term smokers, the patients in these clinical trials were not exposed to nicotine until after PD symptoms developed, a point at which significant dopaminergic neuron loss has already occurred [30].

Here, we tested the hypothesis that there is a timing requirement, or therapeutic window, for nicotine to be effective in the homozygous *park*^*25*^ PD model fly. The homozygous *park*^*25*^ fly is a better model for PD than the heterozygous *park*^*25*^ fly because it has more severe PD-like phenotypes. For example, homozygous *park*^*25*^ flies exhibit dopaminergic neuron loss while the heterozygous *park*^*25*^ flies do not [13, 15]. To do this, we developed a new climbing assay that provides a more detailed analysis than the commonly used negative geotaxis assay [13, 20]. This assay reveals a variety of climbing deficits in homozygous *park*^*25*^ mutants. Like the heterozygous *park*^*25*^ mutants, nicotine benefits both the climbing and flight deficits in these homozygous mutant flies when the nicotine is given from the same day that they eclose from the pupal case (day 0). In support of our hypothesis, we determined that the therapeutic benefits on climbing from nicotine are only present when given from day 0. Furthermore, the therapeutic window for flight was larger, reaching out to day 5. The results suggest that there is a therapeutic window of effectiveness for nicotine in this PD model fly. The potential translation of these findings to PD patients would support the idea that nicotine must be given early in the course of the disease to have beneficial effects, possibly explaining the lack of therapeutic effectiveness observed in the clinical trials.

## 2. Materials and Methods

### 2.1. Drosophila Stocks and Maintenance

Flies with the *park*^*25*^ allele were provided courtesy of Dr. Leo Pallanck from the University of Washington. This allele was generated in a *w*^*1118*^ background, thus, *w*^*1118*^ flies, obtained from the Bloomington *Drosophila* Stock Center (Indiana University), were used as a control population for the *park*^*25*^ mutation [14]. The *park*^*25*^ flies were backcrossed with the *w*^*1118*^ stock to ensure that the *park*^*25*^ flies had all *w*^*1118*^ chromosomes, generating a *w*^*1118*^; *park*^*25*^/TM6C stock. Homozygous *park*^*25*^ pupae were identified and collected into separate collection vials with fresh food to allow for sorting of flies without anesthesia. All flies were initially collected and maintained on a standard cornmeal, molasses, and agar food at 25°C.

### 2.2. Nicotine Treatment

Newly eclosed flies, males and females, were collected every 24 hours, which were considered day 0 flies. A freshly made stock solution of 10 mg/mL (−)-Nicotine (Sigma-Aldrich, Saint Louis, MO) was added to the food after cooling to 70°C to make the final nicotine concentration be 3, 4.5, 6, or 9 *μ*g/mL. The 3 and 4.5 *μ*g/mL concentrations were similar to the 9 and 12 *μ*g/mL concentrations from our previous study, since this nicotine was not the hemisulfate salt [13]. Nicotine food was made fresh every two days and flies were started on nicotine food on days 0–2 for climbing and 0–8 for flight. Behavioral assays were performed on 20-day-old male and female flies, except for the initial time course experiment, where the climbing assay was performed on days 0, 5, 10, and 20. Flies were transferred every 2-3 days onto new vials, with or without nicotine, until the day of the assay. For climbing, the flies were always transferred to new food 24 hours prior to the assay to reduce grooming behavior during the assay.

### 2.3. Climbing Assay

The newly developed climbing assay consisted of using a MultiBeam Activity Monitor (MBM, Trikinetics Inc. Waltham, MA) in a vertical position to measure the flies' climbing ability. The activity monitor can hold and monitor 16 independent activity tubes. The MBM has 9 independent infrared beams for each tube that can detect activity along a 51 mm tube length in 3 mm increments, for a total of 17 unique positions. Individual flies are loaded into a 5 mm × 80 mm polycarbonate, activity tube via an individual fly collection apparatus. This apparatus consists of a 1 mL pipette tip, which has been modified to have a larger orifice, connected to a 0.5 m PVC tube. An individual fly is vacuumed into the pipette tip and then expelled into the locomotion tube by a puff of air. An 18 mm piece of white yarn was placed into the top end of the locomotion tube while another piece was placed 11 mm into the bottom end of the polycarbonate tube to keep the fly constantly in the infrared detection zone of the tube. Each tube was then placed vertically in the MBM. Both *park*^*25*^ homozygotes and control groups were assayed at 20 days of age, unless specified differently. Typically, eight homozygous *park*^*25*^ flies and eight control flies were assayed simultaneously. Climbing was assayed by recording the fly's position every second for 20 minutes.

### 2.4. Climbing Metrics

Various climbing metrics were assessed from the raw data collected from the MBM utilizing an Excel® spreadsheet with macro programming (available upon request from the corresponding author). Many of the climbing metrics are self-explanatory, however, a brief definition of some of them are provided here. A “climb” is counted when a fly reverses travel from climbing up to climbing down. A complete ascent is when the fly would start at the bottom of the tube and climb to the top (51 mm) without reversing direction. The climbing tube was divided into three, 17 mm, equidistant sections: a lower position (1–17 mm), a middle position (18–34 mm), or a higher position (35–51 mm). From this division, the average time spent in each tube position could be obtained. This metric was termed “dwell time.” Movements were defined by any positional change that was captured from second-to-second.

Videos of five *w*^*1118*^ and five homozygous *park*^*25*^ 20-day-old flies were captured at 750 frames/second while they were in the MBM climbing assay for 10 minutes. The MBM climbing data was aligned with the video to allow for the visual identification of whether a descent movement was a drop or a descending climb. Programming in the Excel® climbing template was performed to account for the speed and distance of the descent to allow for the correct classification between a drop or a descending climb. Both types of descents had to be >3 mm (the minimal distance recordable in the MBM). Drops were any descents with a velocity >8.97 mm/sec and descending climbs were a velocity <6 mm/sec. As is apparent, not all descents were able to be classified by the programming, and therefore were excluded from the drop vs. descending climb identification. However, of those that could be differentiated, 298 out of 314 (94.9%) were correctly identified from the MBM data by the programming for the ten videos, indicating a high degree of accuracy. No significant differences in the number of correctly identified descents were found between the *park*^*25*^ and the *w*^*1118*^ flies (92.6% vs. 95.8%, respectively; *t*-test, *P*=0.799).

### 2.5. Flight Assay

Flight capability was always tested in 20-day-old flies. The assay was performed as previously described [13]. Briefly, the flies were tested in a clear acrylic box measuring 28 cm on each side with a 3 cm diameter hole at the top. Individual flies were dropped in from the opening while being covered with a 30 mm Petri dish. If a fly maintained elevation, it was counted as capable of flight; otherwise, it was classified as incapable of flight. Interrater reliability tests have been performed proving 85–93% reliability for this assay. Most flight assays were performed on flies that were previously tested for climbing.

### 2.6. Statistics

Climbing data was analyzed in Prism 9.0 with two-way ANOVA, followed by Tukey's post hoc test to determine the differences. For the flight data, the percentage able to fly for each trial was determined for each treatment group and analyzed using a Chi-Square or Fischer's Exact Test analysis in Excel or Prism. When there were more than two samples to compare, a Marascuillo Chi-Square procedure with a Bonferroni correction was used to determine the significance comparing to the control. All climbing graphs display the mean ± the standard error of the mean. Details on each test performed and their results are presented in the results section or legends.

## 3. Results

### 3.1. Homozygous *park*^*25*^ Flies Demonstrate Climbing Deficits as Early as Day 0 and Maintain These Deficits Through Day 20

**Figure 1:**
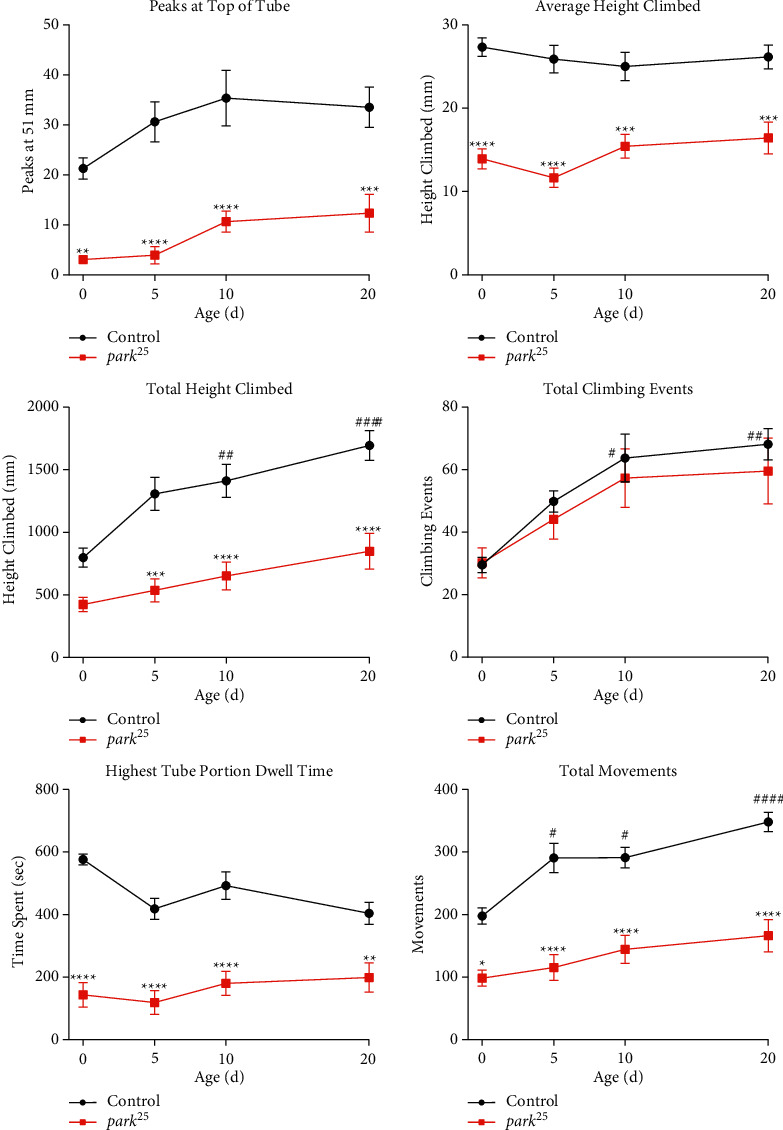
Time course of climbing deficits in homozygous *park*^*25*^ flies. Control and homozygous *park*^*25*^ mutants were collected and aged to 0, 5, 10, and 20 days and assayed in the MBM climbing assay. Different climbing metrics are shown, see Methods for definitions. Data are presented as mean and SEM. Results from a post hoc Tukey's HSD analysis are shown: Asterisks represent comparisons between genotypes, ^*∗∗∗∗*^=*P* < 0.0001, ^*∗∗∗*^=*P* < 0.001, ^*∗∗*^=*P* < 0.01^*∗*^=*P* < 0.05, pound signs represent comparisons within genotypes, ^###^ = *P* < 0.001, ^##^ = *P* < 0.01^#^ = *P* < 0.05. The results of each data point are from three separate experiments with an n ≥ 21 flies.

Since our previous study analyzed climbing ability in heterozygous *park*^*25*^ flies in a simple negative geotaxis assay, we set out to fully characterize the climbing deficits in homozygous *park*^*25*^ flies in our new climbing assay [13]. The average number of climbs that reach 51 mm (the top of the climbing tube) was lower in 0-day-old *park*^*25*^ homozygous flies compared to 0-day-old control flies and this deficit is maintained through day 20 (all ages, *P* < 0.01) (Figure 1). This indicates that *park*^*25*^ homozygotes do not climb to the top of the tube as much or as often as control flies. In agreement with this, the average height climbed in each climbing event was lower in *park*^*25*^ homozygotes versus control flies (all ages, *P* < 0.001). When the height climbed is totaled for the entire 20-minute period, the homozygous *park*^*25*^ flies were lower on days 5–20 (all three ages, *P* < 0.001), although it appears that even day 0 *park*^*25*^ flies climbed less, the difference was not significant. Control flies increase the number of climbing events from day 0 to day 10 (*P* < 0.05) and day 20 (*P* < 0.01). This same pattern is observed in the *park*^*25*^ mutants, in fact, there were no differences in the number of climbing events between the *park*^*25*^ and control flies (all ages, *P* > 0.99). This indicates that homozygous *park*^*25*^ flies make just as many attempts to climb as control flies. However, though the *park*^*25*^ homozygotes are climbing, they do not reach the top portion of the climbing tube as much as the control flies within the 20-minute assay. To measure this, the average time spent by the flies in the top third of the climbing tube (from 35 mm to 51 mm) was measured. On average, *park*^*25*^ homozygous flies spend a lower amount of time in the top portion of the climbing tube compared to the control flies (all ages, *P* < 0.01). Conversely, the *park*^*25*^ homozygotes spend more time on day 0 and 5 in the lower third of the climbing vial when compared to the control flies (Supplemental Figure 1, both *P* < 0.001). It appears that the *park*^*25*^ homozygotes try to climb, but fail in maintaining their climbing, spending more time in the bottom of the tube. In support of this, the average height reached in the climbing tube for each climbing event (average peak height) was lower at all ages in *park*^*25*^ mutants compared to controls (Supplemental Figure 1, all ages, *P* < 0.001). Homozygous *park*^*25*^ flies are less active at all ages (all ages, *P* < 0.05), as measured by total movements. Though they move less, their ascending velocity is not different from controls (Supplemental Figure 1, all ages, *P* > 0.12), possibly indicating that their physical ability to climb is not reduced. Though the ascending velocity is not different, the descending velocity of day 0 *park*^*25*^ flies is greater than the control flies (Supplemental Figure 1, *P* < 0.0001). Why these young flies descend faster, is unknown, but perhaps one explanation could be that instead of climbing down, they were actually falling down, which could lead to a faster velocity measurement. As a final measurement of climbing ability, the number complete ascents were determined. This is a measure of the ability of flies to climb the entire length of the climbing tube from the bottom to the top without reversing direction at any time in the climb. This pattern of climbing is likely what all flies desire to do but do the most infrequently. Except for the 10-day-old flies, the *park*^*25*^ mutants made less complete ascents compared to control flies (Supplemental Figure 1, remaining three ages, *P* < 0.05). Overall, it is apparent that homozygous *park*^*25*^ flies have more significant climbing deficits compared to our previous study in heterozygous flies [13] that occur at earlier ages, typically starting at the time of eclosion.

### 3.2. Nicotine Improves Climbing Deficits in Homozygous *park*^*25*^ Flies When Given on Day 0

**Figure 2:**
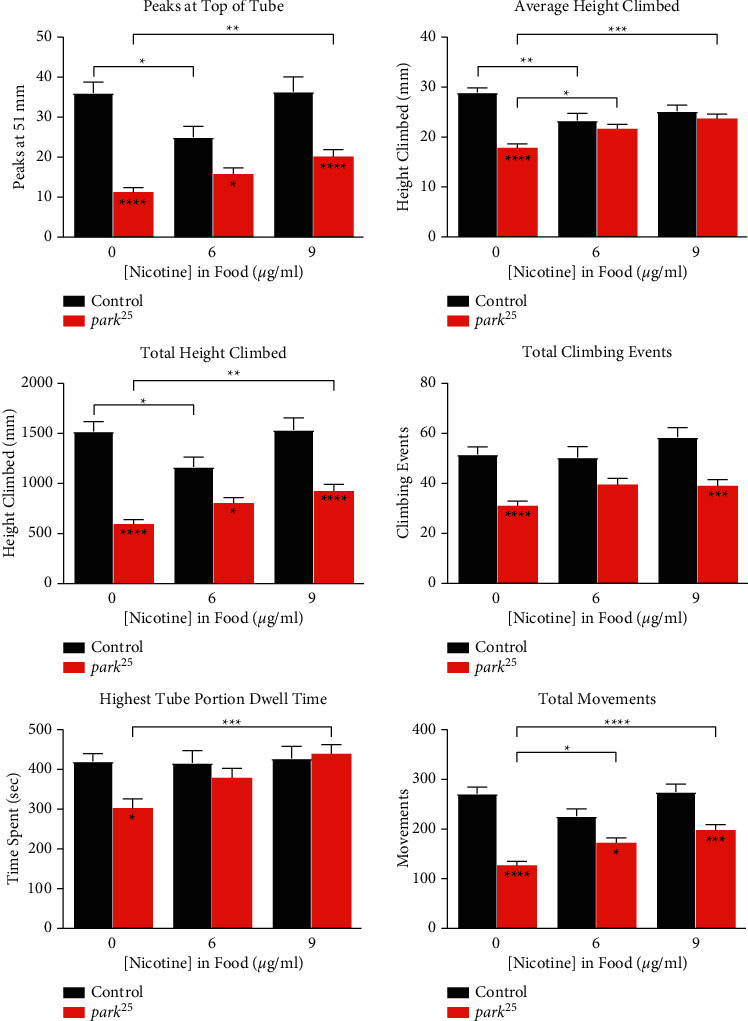
Nicotine treatment improves climbing deficits in *park*^*25*^ homozygotes. Nicotine (6 and 9 *μ*g/mL) was given to homozygous *park*^*25*^ flies compared to control flies from day 0. Different climbing metrics are shown, see Methods for definitions. Data are presented as mean and SEM. Results from a post hoc Tukey HSD analysis are shown. Asterisks inside the bars compare between the two genotypes at individual nicotine levels. ^*∗∗∗∗*^=*P* < 0.0001, ^*∗∗∗*^=*P* < 0.001, ^*∗∗*^=*P* < 0.01^*∗*^=*P* < 0.05. The results of each data point are from at least four separate experiments with an n ≥ 56 flies.

We have previously determined that nicotine treatment at 3 and 4.5 *μ*g/mL improved climbing ability in heterozygous *park*^*25*^ flies [13]. Therefore, we set out to determine if the same beneficial nicotine effects can be observed in homozygous *park*^*25*^ flies. In all previously measured climbing metrics, 9 *μ*g/mL of nicotine, when given from day 0, improved the homozygous *park*^*25*^ mutant climbing deficit (Figure 2 and Supplementary Figure 2, all *P* < 0.01), except for total climbing events, and both ascending and descending velocity. In this set of experiments, the *park*^*25*^ mutants have a decreased number of climbing events compared to control flies (*P* < 0.0001), which was not observed in the 20-day-old flies (Figure 1). Nevertheless, nicotine did not affect the number of climbing events, which again points to this climbing metric different from the rest. Indeed, this climbing metric may be more of a CNS phenomenon, measuring the flies' motivation to climb.

While 6 *μ*g/mL of nicotine did improve some of the climbing deficits in the *park*^*25*^ mutants, it was not as effective as 9 *μ*g/mL. This is interesting because these doses are double the effective doses used in our previous heterozygous *park*^*25*^ study [13], reinforcing the idea that the homozygous *park*^*25*^ phenotypes are more severe and take a greater amount of nicotine to overcome. In agreement with this, 3 and 4.5 *μ*g/mL of nicotine, which were equivalent to the previously used nicotine concentrations, had no effect in the *park*^*25*^ homozygotes except in complete ascents (Supplemental Figure 3).

Another observed nicotine effect that replicated our previous findings was the overall negative effect of nicotine on the control flies. While nicotine tended to improve climbing performance in the *park*^*25*^ mutants, it had a detrimental effect in many of the climbing metrics in the control flies. This was even observed at the lower (3 and 4.5 *μ*g/mL) amounts (Supplemental Figure 3).

### 3.3. Nicotine Improves Flight Ability in Homozygous park^25^ Flies

**Figure 3:**
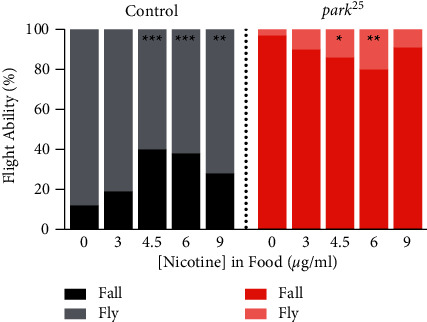
Nicotine increases flight ability in homozygous *park*^*25*^ flies. Homozygous *park*^*25*^ and control flies were tested for their ability to fly with various concentrations of nicotine from day 0. Results from a post hoc Marascuilo procedure of a Chi-square analysis with a Bonferroni correction are shown. Asterisks inside the bars compare between that group and the 0 *μ*g/mL group within that fly genotype. ^*∗∗∗*^=*P* < 0.001, ^*∗∗*^=*P* < 0.01^*∗*^=*P* < 0.05. The results of each data point are from at least four separate experiments with an n ≥ 50 flies.

The vast majority (93%) of the untreated homozygous *park*^*25*^ flies were unable to fly in the flight assay, compared to the 88% of control flies that flew (Figure 3; *n*: 99 *park*^*25*^, 154 control). Both 4.5 and 6 *μ*g/mL concentrations of nicotine, when given from day 0, improved the flight ability of the *park*^*25*^ mutants (*P* < 0.05), although the maximal percentage of flying was only a modest 20% with 6 *μ*g/mL of nicotine. Nicotine had a negative effect on the control flies, with flight being reduced by all concentrations above 3 *μ*g/mL (*P* < 0.01).

### 3.4. Nicotine Does Not Improve Climbing Deficits in *park*^*25*^ Flies When Given after Day 0

**Figure 4:**
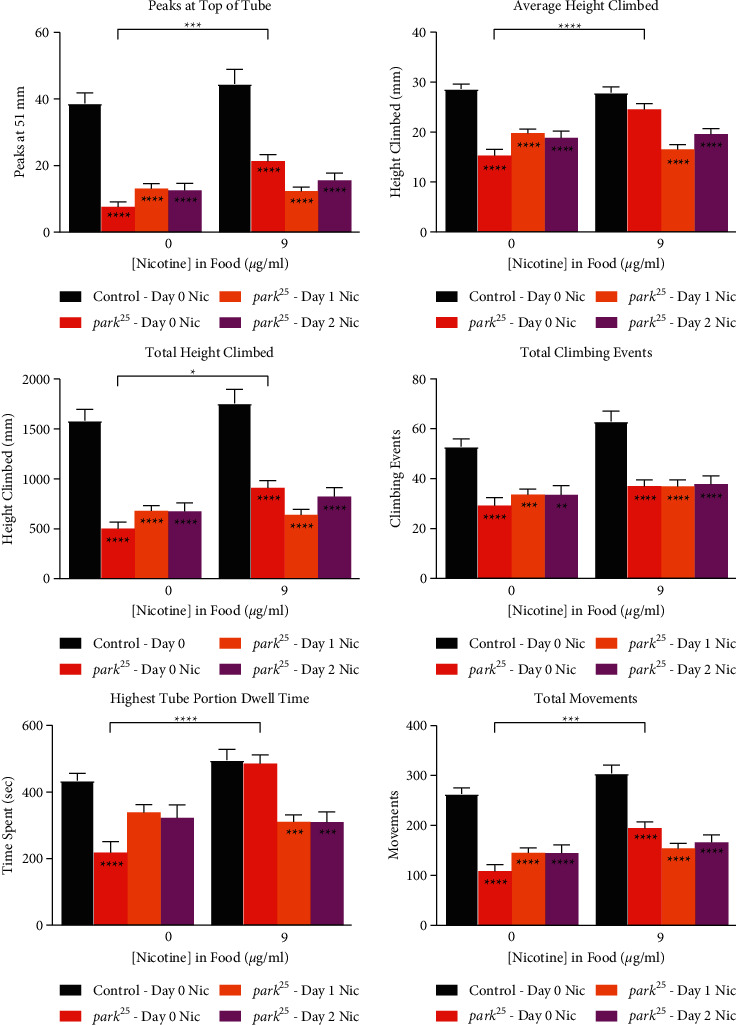
Delayed nicotine does not provide benefits in climbing in homozygous *park*^*25*^ flies. Nicotine (9 *μ*g/mL) was given to homozygous *park*^*25*^ and control flies on day 0 post-eclosion as well as on days 1 and 2 post-eclosion to the *park*^*25*^ flies. Different climbing metrics are shown, see Methods for definitions. Data are presented as mean and SEM. Results from a post hoc Tukey HSD analysis are shown. Asterisks inside the bars compare between the two genotypes at individual nicotine levels. ^*∗∗∗∗*^=*P* < 0.0001, ^*∗∗∗*^=*P* < 0.001, ^*∗∗*^=*P* < 0.01^*∗*^=*P* < 0.05. The results of each data point are from at least four separate experiments with an n ≥ 38 flies.

Given the improvements in climbing performance due to nicotine given on day 0 post-eclosion, we sought to determine if the delayed administration of nicotine could also provide these beneficial effects. Nicotine (9 *μ*g/mL) was initiated on days 0–2 post-eclosion and then maintained until the climbing assay. When administration of nicotine was delayed by one or two days, all improvements in climbing were absent (Figure 4 and Supplemental Figure 4).

### 3.5. Nicotine Improves Flight Deficits When Given on Days 0–5 Post-Eclosion

**Figure 5:**
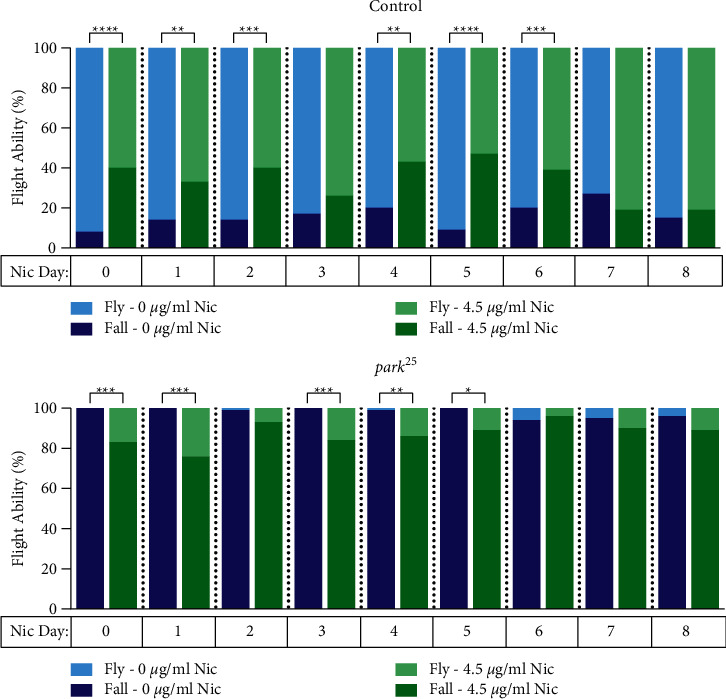
Nicotine (4.5 *μ*g/mL) improves flight performance in homozygous *park*^*25*^ flies when given on days 0–5 post-eclosion. Control flies and *park*^*25*^ mutants were initiated with 4.5 *μ*g/mL nicotine on different starting days (days 0–8) post-eclosion. On day 20, flies were tested for their ability to fly. Results from individual Fisher's Exact Tests between the control and *park*^*25*^ flies at each nicotine concentration are shown. ^*∗∗∗∗*^=*P* < 0.0001, ^*∗∗∗*^=*P* < 0.001, ^*∗∗*^=*P* < 0.01^*∗*^=*P* < 0.05. The results of each data point are from at least five separate experiments with an n ≥ 36 flies (see Supplemental Table 1 for details).

Since nicotine, at 4.5 *μ*g/mL, when given on day 0 improved flight performance in homozygous *park*^*25*^ flies, a time course of delayed initiation of nicotine was performed from days 0–8 post-eclosion in homozygous *park*^*25*^ and control flies. Nicotine improved flight performance in the first five days of the time course in the *park*^*25*^ mutants (Figure 5). A similar effect by nicotine was also observed with the control flies, but in the opposite direction; nicotine decreased flight performance in the control flies through day six post-eclosion. The same overall results were observed when 3 *μ*g/mL of nicotine was given to the flies across the eight-day initiation period, with the homozygous *park*^*25*^ flies benefitting and the control flies being negatively affected through day five by nicotine (Supplemental Figure 5). Large numbers of flies were tested on days 6–8 to ensure that the effects of nicotine were truly lost by then (Supplemental Table 1).

### 3.6. Nicotine Improves the Quality of the Descent in the *park*^*25*^ Fly

**Figure 6:**
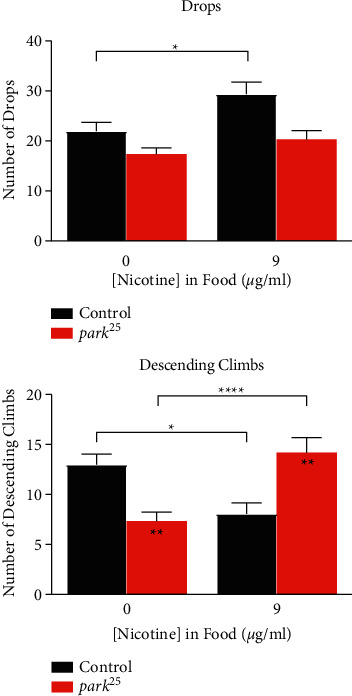
Nicotine treatment increases the number descending climbs in homozygous *park*^*25*^ flies. Number of (a) drops or (b) descending climbs (see Methods for details) in nicotine-treated flies. Data are presented as mean and SEM. Results from a post hoc Tukey HSD analysis are shown. Asterisks inside the bars compare between the two genotypes at individual nicotine levels. ^*∗∗∗∗*^=*P* < 0.0001, ^*∗∗*^=*P* < 0.01^*∗*^=*P* < 0.05. The results of each data point are from at least four separate experiments with an n ≥ 56 flies.

We sought to determine a mechanism behind the improvements in climbing with nicotine treatment in the homozygous *park*^*25*^ flies. High speed video of ten flies (five *park*^*25*^ and five control) was captured while simultaneously performing the MBM climbing assay for 10 minutes. This allowed for the programmatic detection of two different types of descents in the climbing assay: falls and descending climbs (see Methods for more details). Analysis of the same climbing data from Figure 2 reveals that control flies have more descending climbs than the *park*^*25*^ mutants (*P* < 0.0022), while they drop at equal rates (*P*=0.1871) in the entire 20-minute climbing period (Figure 6). Nicotine (9 *μ*g/mL) treatment increased the number of descending climbs in the homozygous *park*^*25*^ flies (*P* < 0.0001). Nicotine had the opposite effect in control flies by decreasing the number of descending climbs (*P*=0.0444) and increasing the number of drops (*P*=0.0372). Similar results were observed in an overall percentage analysis of the descent data (data not shown), since there are descents that are not classifiable by the programming.

## 4. Discussion

This study set out to help determine why there is a discrepancy between PD models and PD patients regarding nicotine treatment. The epidemiological evidence of smokers having reduced incidence of PD is one of the most robust correlations found between PD and the environment [23, 25, 31–34]. These findings likely prompted clinical trials administering nicotine to PD patients, which had mixed results [35–37]. In contrast, nicotine's beneficial effects in multiple PD models are well recognized [22]. In *Drosophila,* specifically, nicotine has been shown to benefit heterozygous *park*^*25*^ flies and another PD model fly [13, 20]; however, these studies administered nicotine from day 0. This study determined that nicotine benefits the homozygous *park*^*25*^ PD model fly and has a discrete therapeutic window for that effect. A similar finding has been found in a rat nigrostriatal damage PD model [24]. Rats that received nicotine both before and immediately following lesioning had improved behavioral deficits from lesion-induced losses of striatal dopaminergic neurons. However, rats that received nicotine two weeks post-lesioning did not improve by the same degree [24]. Similar results were found in another study in lesioned rats and monkeys [38]. These studies support our results that nicotine's benefits have a window of effectiveness.

Nicotine's therapeutic window may be due to advanced dopaminergic neuron loss. It has been shown that nicotine can be neuroprotective [25, 39, 40], but, if a significant amount of neurons have already been lost, it is likely that nicotine can have no meaningful benefits. This could explain the clinical trial results, since it is estimated that by the time of diagnosis, PD patients have already experienced significant (30%–50%) dopaminergic neuron loss [30]. Dopaminergic neuron loss has been documented in *park*^*25*^ homozygous flies, with loss of about 1-2 neurons (∼8%–17%) on day 1, and about 4 (∼33%) by day 20 [15]. Therefore, in our model, perhaps day 0 nicotine administration is before any significant neuronal loss occurs, but after that, neural degeneration has begun. This may explain why nicotine loses its efficacy if exposure begins after day 1 for climbing and after day 5 for flight.

Our data clearly demonstrate that climbing and flight both have therapeutic windows for nicotine; however, there are some differences between these two systems. Most strikingly, the length of the window for administration is much shorter in climbing, one vs. five days (Figures 4 and 5). Next, the concentration of nicotine to have an effect is lower for flight than it is for climbing, 3 *μ*g/mL vs. 6 *μ*g/mL (Figure 2 and Supplemental Figures 3 and 5). Interestingly, the timing and dose of nicotine required to produce benefits in the homozygous *park*^*25*^ flies were very similar to the dose and timing of nicotine that produced detrimental effects in control flies. This suggests that nicotine may be producing these effects by different mechanisms. It is important to note that acetylcholine, via nicotinic receptors, is not the mechanism by which neuromuscular junctions operate in *Drosophila*. Rather, glutamate is the neurotransmitter in the neuromuscular junction [41]. Therefore, nicotine's effects in flies are not directly on the neuromuscular junction but must be through an indirect mechanism. Perhaps, the differences between these two systems are a result of receptor-mediated and non-receptor-mediated effects by nicotine [23, 37]. Indeed, unpublished data from our laboratory with heterozygous *park*^*25*^ flies suggest that this might be the case.

To better understand motor deficiencies in PD model flies, more robust locomotor assays have recently been developed to replace the commonly used negative geotaxis assay [42, 43]. While our new climbing assay has been briefly reported on before for obtaining average height climbed [16], our current study presents our newly developed climbing assay in full detail. This climbing assay can reveal multiple locomotory behaviors, which can provide further insight into the *park*^*25*^ mutant phenotype. It has been well documented that both homozygous and heterozygous *park*^*25*^ flies have deficiencies in climbing [13, 14], but now we understand that *park*^*25*^ flies climb less each time they climb, but at a similar or lower velocity as control flies (Figures 1 and 2; Supplemental Figures 1 and 2). It is interesting that the number of climbing events was not different between the control and *park*^*25*^ flies in the time course experiment (Figure 1), although it was reduced in the nicotine experiment (Figure 2). This metric only counts the number of times the flies climbed, not how far or well they climbed. Therefore, this metric might be more of a measurement of the motivation to climb, which has been reported to be reduced in a severe PD model fly [42]. Additionally, because the *park*^*25*^ mutants do not climb as high, they do not spend as much time in the top of the tube as the control flies (Figures 1 and 2), which has an inverse relationship with the time spent at the bottom of the tube (Supplemental Figures 1 and 2). Analysis of the drop vs. descending climb behavior revealed that while both control and *park*^*25*^ flies drop in the tubes similarly, *park*^*25*^ flies performed fewer descending climbs than controls (Figure 6). However, nicotine treatment increased the descending climb numbers in the *park*^*25*^ mutants to untreated control levels, suggesting that nicotine improved the ability of the *park*^*25*^ fly to stay attached to the tube. Nicotine had the opposite effect in the control flies, causing them to drop more and reducing the number of descending climbs. This negative effect of nicotine on the control flies was a continual pattern across all the assays. This is very intriguing, as nicotine is beneficial in *park*^*25*^ flies and detrimental in the control flies. This is similar to what was observed with the heterozygous *park*^*25*^ flies [13]. In fact, many of the deficits observed in the homozygous *park*^*25*^ flies were very similar to that observed in heterozygous *park*^*25*^ flies, only more severe, both in amount and age of onset.

This study adds to a limited number of studies in differing PD models that indicate that nicotine has a therapeutic window of effectiveness. Our approach distinctly shows that after a certain time point, nicotine's beneficial effects are completely lost in our model, both for flight (after five days) and climbing (after one day). As with any model, there are limitations in translating the results to humans. Indeed, all PD models are not perfect; however, the fruit fly has been a robust and useful PD model, contributing much understanding to the PD field [12, 44–48]. This study definitively demonstrates a therapeutic window of effectiveness for nicotine in the *park*^*25*^ homozygous fly. Given this, it may help explain why there were mixed results when PD patients were administered nicotine. Perhaps if nicotine is administered earlier to PD patients, nicotine may be able to provide more beneficial effects. Whether this is possible with the current diagnostic approaches for PD is unknown, however, immediately treating newly diagnosed PD patients with nicotine might be an approach that would help to address this important question. If this treatment is still found to be ineffective, earlier diagnostic approaches may be the solution, like olfaction loss or peripheral *α*-synuclein detection [49–51]. It is hoped that this study, in conjunction with the previous studies showing similar results in mammals [24, 38], will promote modified clinical trials treating PD patients with nicotine earlier in the disease with the hope of revealing its promised therapeutic potential.

## Data Availability

The datasets used during the present study are available from the corresponding author upon reasonable request.

## Supplementary Materials

Supplementary Materials The supplementary materials report included with this paper contains additional figures and a table.
