# Supplementary material for: Nicotine Has a Therapeutic Window of Effectiveness in a Drosophila melanogaster Model of Parkinson's Disease
Source: Parkinsons Dis. 2022 Jul 8;2022:9291077. doi: 10.1155/2022/9291077 (PMC9286976; doi:10.1155/2022/9291077)
Supplement: Supplementary Materials — The supplementary materials report included with this paper contains additional figures and a table. [file 9291077.f1.pdf]

Supplementary Materials for:

Nicotine has a therapeutic window of effectiveness in a *Drosophila melanogaster* model of Parkinson's disease

Brady T. Mannett[a,b], Braden C. Capt[a], Krista Pearman[c], Lori Buhlman[d], John M. VandenBrooks[e], Gerald B. Call[c*]

[a]Arizona College of Osteopathic Medicine, Midwestern University, Glendale, AZ 85308

[b]Current Address: OSF Neurology at University of Illinois, Peoria, IL 61637

[c]Department of Pharmacology, College of Graduate Studies, Midwestern University, Glendale, AZ 85308

[d]Biomedical Science Program, College of Graduate Studies, Midwestern University, Glendale, AZ 85308

[e]Department of Physiology, College of Graduate Studies, Midwestern University, Glendale, AZ 85308

*Corresponding author: gcall@midwestern.edu

Supplemental Table 1. Number of flies tested in delayed nicotine (4.5 µg/mL) experiments.

**Start day**	0		1		2		3		4		5		6		7		8	
**[Nicotine]**	0	9	0	9	0	9	0	9	0	9	0	9	0	9	0	9	0	9
**Control flies**	116	115	94	102	83	57	92	68	109	67	58	45	134	179	55	67	78	97
***park*^{25} flies**	79	60	56	38	71	40	69	49	79	44	60	36	114	95	57	68	49	55

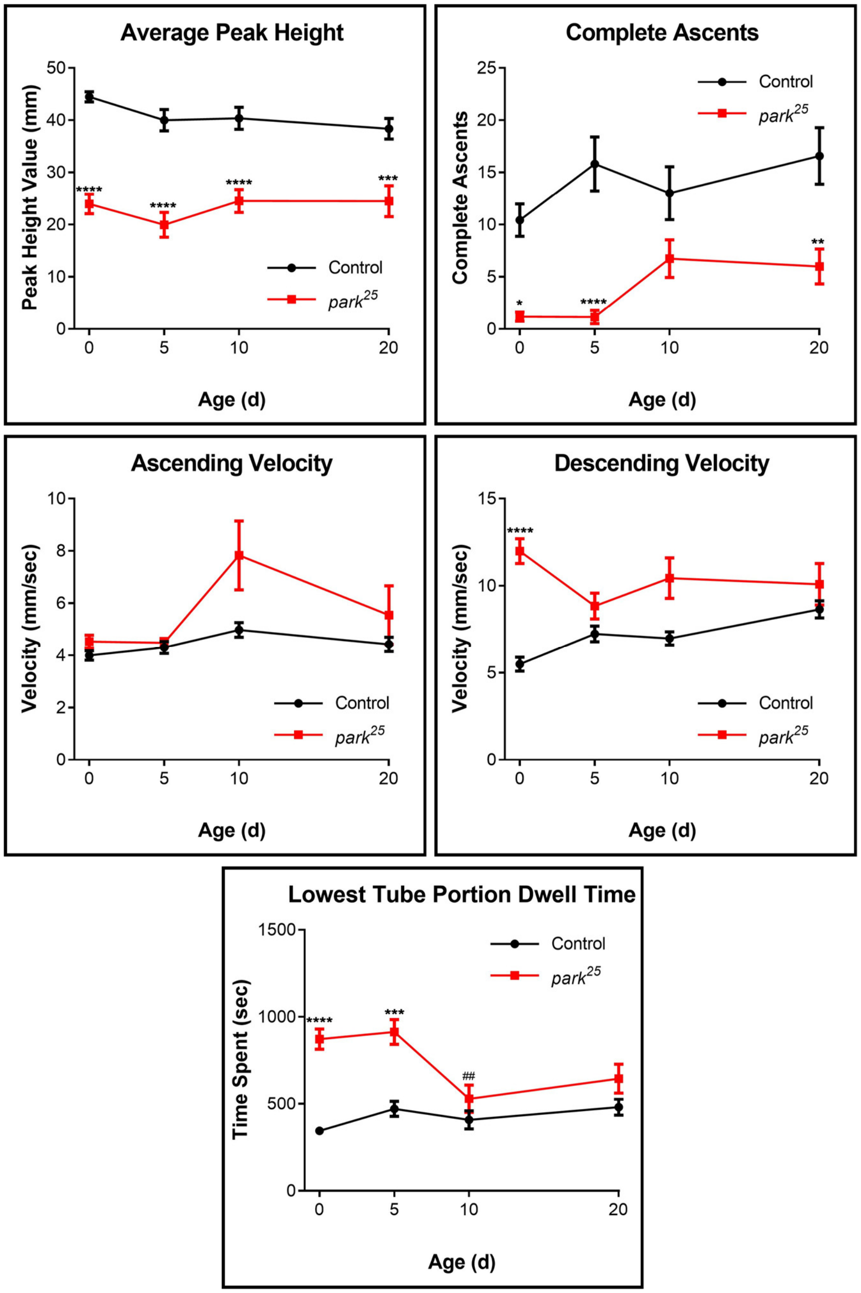

Supplemental Figure 1. Time course of additional climbing deficits in homozygous *park*^{25} flies. Control and homozygous *park*^{25} mutants were collected and aged to 0, 5, 10 and 20 days and assayed in the MBM climbing assay. Different climbing metrics are shown, in addition to those in **Figure 1**, see Methods for definitions. Data are presented as mean and SEM. Results from a post-hoc Tukey's HSD analysis are shown: Asterisks represent comparisons between genotypes, **** = $P < 0.0001$, *** = $P < 0.001$, ** = $P < 0.01$ * = $P < 0.05$, pound signs represent comparisons within genotypes, ## = $P < 0.01$. The results of each data point are from three separate experiments with an $n \geq 21$ flies.

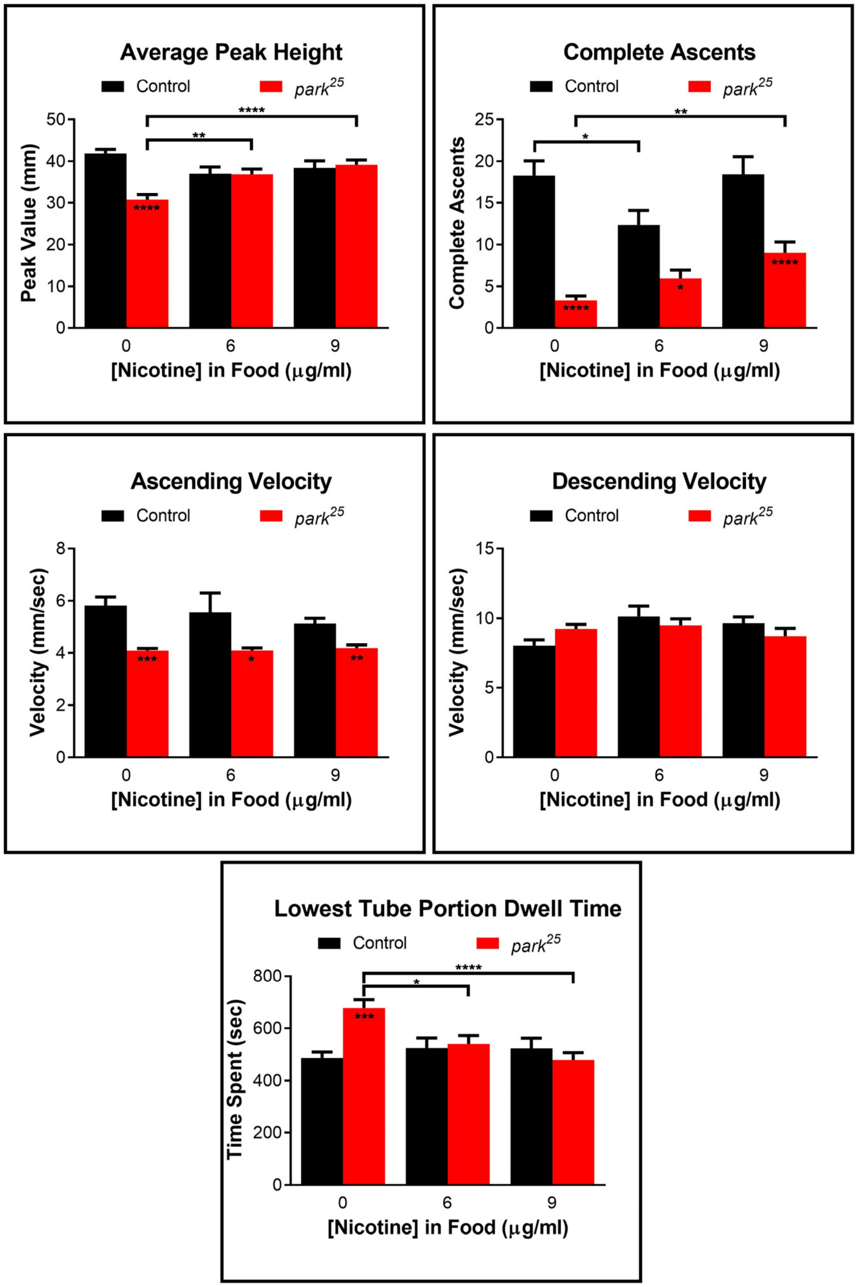

Supplemental Figure 2. Nicotine treatment improves additional climbing deficits in *park*^{25} homozygotes.
Nicotine (6 and 9 µg/mL) was given to homozygous *park*^{25} flies compared to control flies from day 0. Different climbing metrics are shown, in addition to those in **Figure 2**, see Methods for definitions. Data are presented as mean and SEM. Results from a post-hoc Tukey HSD analysis are shown. Asterisks inside the bars compare between the two genotypes at individual nicotine levels. **** = P < 0.0001, *** = P < 0.001, ** = P < 0.01 * = P < 0.05. The results of each data point are from at least four separate experiments with an *n* ≥ 56 flies.

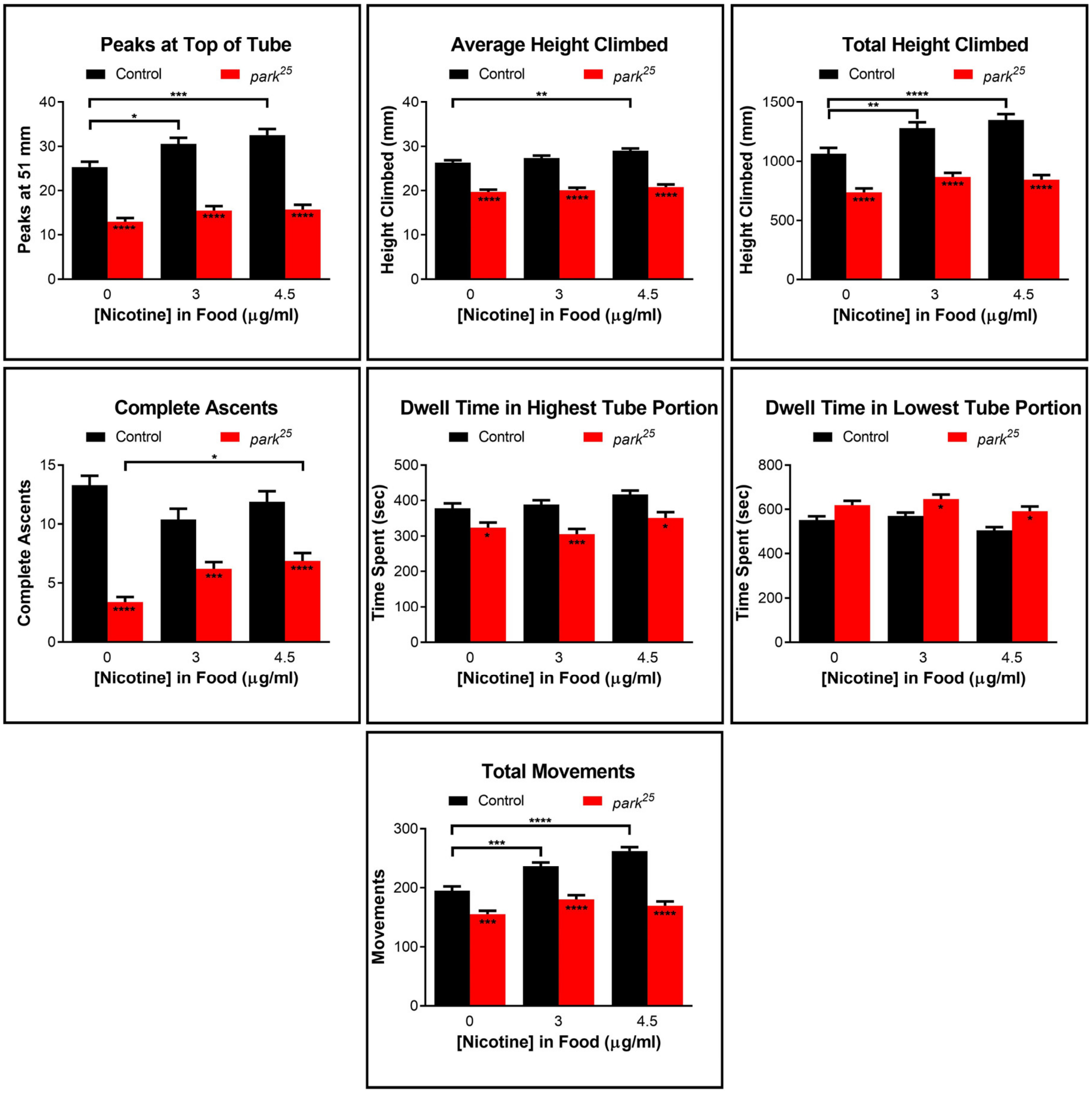

Supplemental Figure 3. Reduced nicotine levels are not as effective at improving climbing deficits in homozygous *park*^{25} mutants.

Nicotine (3 and 4.5 μg/mL) improves only the deficit in complete ascents present in homozygous *park*^{25} flies compared to control flies. Other, non-improved climbing metrics are shown, see Methods for definitions. Data are presented as mean and SEM. Results from a post-hoc Tukey HSD analysis are shown. Asterisks inside the bars compare between the two genotypes at individual nicotine levels. **** = P < 0.0001, *** = P < 0.001, ** = P < 0.01 * = P < 0.05. The results of each data point are from at least 21 separate experiments with an *n* ≥ 220 flies.

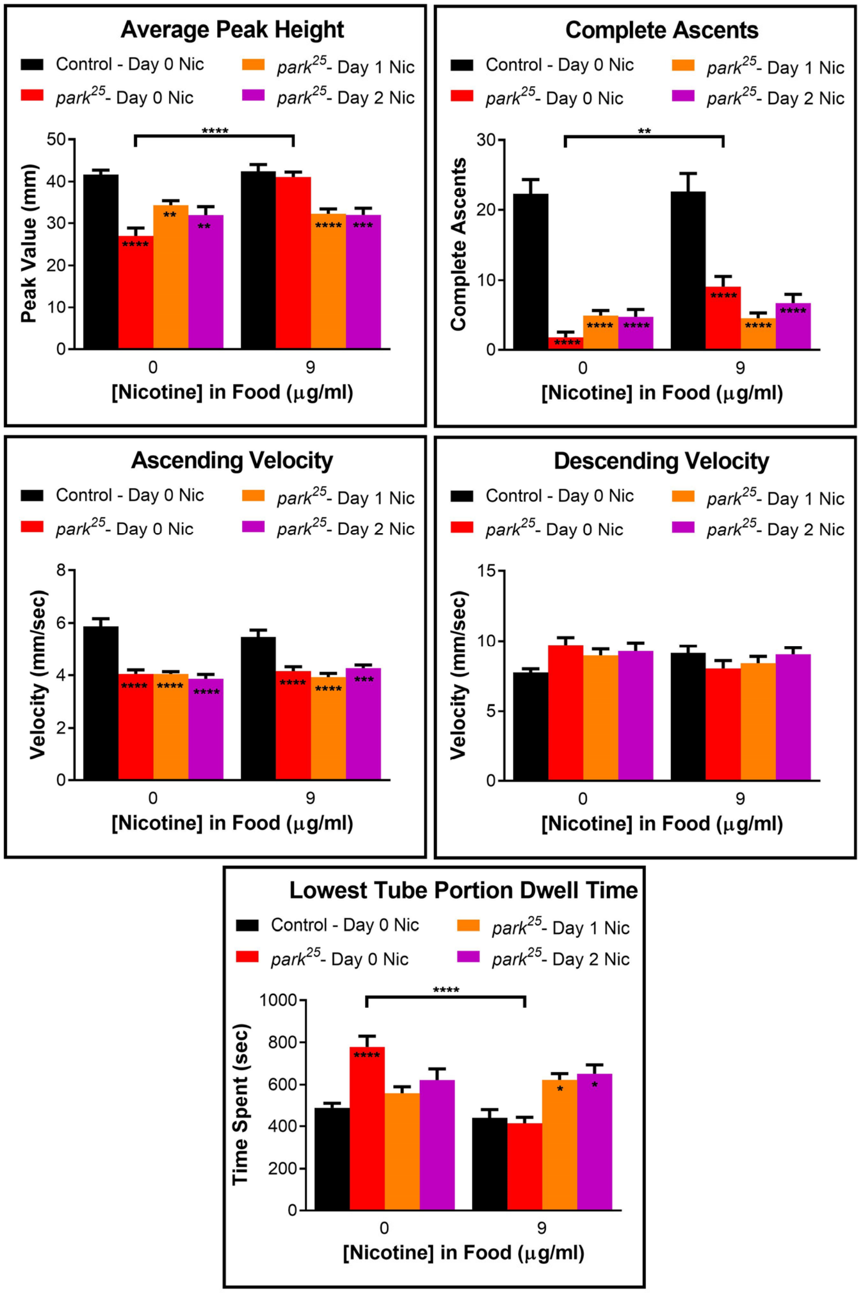

Supplemental Figure 4. Delayed nicotine does not have beneficial effects in other climbing metrics. Nicotine (9 µg/mL) was given to homozygous *park*^{25} and control flies on day 0 post-eclosion as well as on days 1 and 2 post-eclosion to the *park*^{25} flies. Different climbing metrics are shown, in addition to those in **Figure 4**, see Methods for definitions. Data are presented as mean and SEM. Results from a post-hoc Tukey HSD analysis are shown. Asterisks inside the bars compare between the two genotypes at individual nicotine levels. **** = P < 0.0001, *** = P < 0.001, ** = P < 0.01 * = P < 0.05. The results of each data point are from at least four separate experiments with an *n* ≥ 38 flies.

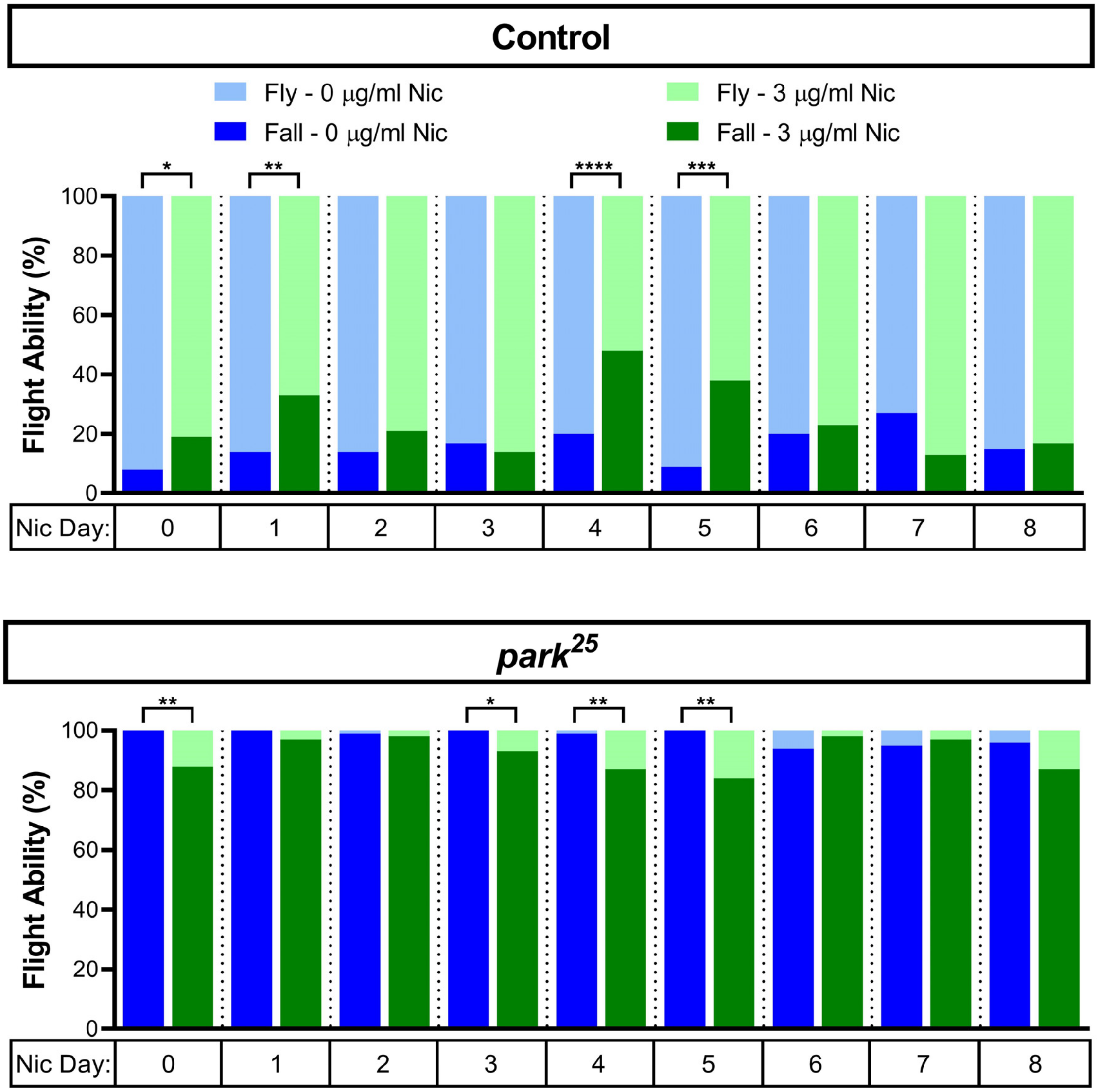

Supplemental Figure 5. Delayed nicotine at 3 µg/mL is beneficial from days 0-5 for flight in *park*^{25} homozygous flies.

Control and homozygous *park*^{25} flies were initiated with 3 µg/mL nicotine on different starting days (days 0-8) post-eclosion. On day 20, flies were tested for their ability to fly. Results from individual Fisher's Exact Tests between the control and *park*^{25} flies at each nicotine concentration are shown. **** = $P < 0.0001$, *** = $P < 0.001$, ** = $P < 0.01$ * = $P < 0.05$. The results of each data point are from at least five separate experiments with an $n \geq 29$ flies.
